# External Validation of a Machine Learning Model for Schizophrenia Classification

**DOI:** 10.3390/jcm13102970

**Published:** 2024-05-17

**Authors:** Yupeng He, Kenji Sakuma, Taro Kishi, Yuanying Li, Masaaki Matsunaga, Shinichi Tanihara, Nakao Iwata, Atsuhiko Ota

**Affiliations:** 1 Department of Public Health, Fujita Health University School of Medicine, Toyoake 470-1192, Japan; 2 Department of Psychiatry, Fujita Health University School of Medicine, Toyoake 470-1192, Japan; 3 Department of Public Health and Health Systems, Nagoya University Graduate School of Medicine, Nagoya 466-8550, Japan; 4 Department of Public Health, School of Medicine, Kurume University, Kurume 830-0011, Japan

**Keywords:** external validation, schizophrenia, bipolar, depression, machine learning, classification, neural network

## Abstract

**Background and Objective:** Excellent generalizability is the precondition for the widespread practical implementation of machine learning models. In our previous study, we developed the schizophrenia classification model (SZ classifier) to identify potential schizophrenia patients in the Japanese population. The SZ classifier has exhibited impressive performance during internal validation. However, ensuring the robustness and generalizability of the SZ classifier requires external validation across independent sample sets. In this study, we aimed to present an external validation of the SZ classifier using outpatient data. **Methods:** The SZ classifier was trained by using online survey data, which incorporate demographic, health-related, and social comorbidity features. External validation was conducted using an outpatient sample set which is independent from the sample set during the model development phase. The model performance was assessed based on the sensitivity and misclassification rates for schizophrenia, bipolar disorder, and major depression patients. **Results:** The SZ classifier demonstrated a sensitivity of 0.75 when applied to schizophrenia patients. The misclassification rates were 59% and 55% for bipolar disorder and major depression patients, respectively. **Conclusions:** The SZ classifier currently encounters challenges in accurately determining the presence or absence of schizophrenia at the individual level. Prior to widespread practical implementation, enhancements are necessary to bolster the accuracy and diminish the misclassification rates. Despite the current limitations of the model, such as poor specificity for certain psychiatric disorders, there is potential for improvement if including multiple types of psychiatric disorders during model development.

## 1. Introduction

Models trained using machine learning approaches have been applied in various fields of medical research [1,2]. They utilize multiple features to detect or estimate an individual’s existence or likelihood of a medical condition [3]. During the training process, it is essential to evaluate the model’s performance in terms of discriminating individuals who have the outcome from those who do not or to accurately estimate the individual’s risk. This can be tested through internal validation, such as bootstrapping, using the same dataset that was used in the training process. However, before deploying these pretrained models in real-world scenarios, the generalizability should be thoroughly confirmed through external validation. This involves testing the pretrained model using one or more independent sample sets. A model that deserves promotion and can be encapsulated as a tool for commercial use should exhibit excellent generalizability.

From 2021 to 2023, we conducted a research program focused on developing a prevalence estimation method for out-of-hospital schizophrenia and schizophrenia-related disorders in the general population. The program used large-scale epidemiological study data collected from an online survey. It involved information on demographic details, health-related backgrounds, physical and psychiatric comorbidities, and social comorbidities [4]. Based on that information, we developed a machine-learning model for classifying the presence or absence of schizophrenia (SZ classifier), in order to identify potential patients and estimate the prevalence of schizophrenia within the general population. The research design of the program is the first of its kind in Japan. 

We envision that if there is a tool that can accurately detect the presence or absence of schizophrenia, it could contribute to the accurate estimation of the prevalence. However, the exact prevalence among the general population is poorly reported. Charlson et al. estimated the global age-standardized point prevalence of schizophrenia in 2016 to be 0.28%, but the actual prevalence could be higher, considering population growth and aging [5]. In Japan, an age–period–cohort analysis reported that the point prevalence was approximately 0.7% [6]. Challenges in the accurate estimation are considered, as (1) some patients may not know that they have the disease. Those who are sick may not always visit the doctor in time; (2) patients with mild disease may not seek medical attention; and (3) some diagnosed cases are for prescriptions to pass a medical insurance review. Hence, the estimated prevalence based solely on medical institution data may not represent the actual incidence in the population. Detecting these “potential” patients in the general population will be the key to estimating the prevalence.

The SZ classifier has provided a promising solution. In the internal validation, it achieved an area under the receiver operating characteristic curve (AUC) score of 0.86 [7]. This indicates that our SZ classifier successfully captures the differences between patients and healthy controls in demographic details, health-related backgrounds, physical and psychiatric comorbidities, and social comorbidities. For example, compared to healthy controls, individuals with schizophrenia demonstrate poor subjective well-being, happiness, and life satisfaction [8,9]. They are also more likely to have chronic diseases, such as poor oral health, noncommunicable diseases, and sleep disorders [8,9]. They also tend to have a lower socioeconomic status [8,9]. 

The SZ classifier was developed using artificial neural networks. Typically, machine learning validation reports rely on internal validation, employing a subset of the training sample set to assess the model performance. However, this approach carries the risk of inflating the model performance due to overfitting [10]. To accurately evaluate model generalizability, external validation is essential, utilizing an independent sample set [11]. Ideally, the sample set should comprise real-world data, which may provide evidence for medical application [12]. Therefore, in the current study, we aimed to present the external validation results of the SZ classifier. To further assess the external validation, we sampled 150 patients from those who visited the Psychiatric Department of Fujita Health University Hospital. The majority of these patients are from Central Japan, where the hospital is located. We anticipate that the SZ classifier can serve as a tool for detecting “potential” patients within the regional community, thereby allowing for estimating the incidence rate, and the subsequent promotion for broader population-level applications.

## 2. Materials and Methods

### 2.1. Development of the Schizophrenia Classification Model (SZ Classifier)

The SZ classifier was developed in our previous study [7] using a sample set collected from an internet research agency’s pooled panel (Rakuten Insight, Inc., Tokyo, Japan) [13]. It comprised 223 schizophrenia patients and 1776 healthy controls. Each sample’s data comprised the presence of schizophrenia, serving as the response variable, along with 76 features. These features included demographic details, health-related backgrounds, physical and psychiatric comorbidities, and social comorbidities. Details of the inclusion criteria have been published elsewhere [9], and the feature definitions are described in Supplementary Materials. The SZ classifier was constructed based on an artificial neural network, considering the significance of capturing nonlinear relationships and higher-order interactions. Specifically, it was structured with five hidden layers, with 128, 64, 32, 16, and 8 neurons in each layer, respectively. The details of the SZ classifier development have been published elsewhere [7] and described in Supplementary Materials. The model performance was examined using the AUC score. 

In the internal validation results, 20% of the whole samples were left out as the test set to assess the model performance. The SZ classifier achieved an AUC of 0.86, accuracy of 0.93, sensitivity of 0.56, and specificity of 0.96 [7]. Also, the results of 10-fold cross-validation exhibited that models trained by artificial neural networks could achieve an AUC of around 0.81 [7]. 

### 2.2. Samples for External Validation

We targeted patients who visited the Psychiatric Department of Fujita Health University Hospital between January 2022 and May 2023. The patients aged between 20 and 75 years and were diagnosed with schizophrenia, major depressive disorder (MDD), bipolar disorder (BD), or obsessive–compulsive disorder (OCD) were selected. The authors, KS and TK, explained the study’s purpose and methods to the patients. Patients voluntarily decided whether to participate in our study. All the participants provided written informed consent to participate in this study. We confirmed that they had not participated in our research team’s online survey (the aforementioned SZ classifier study). 

Participants were asked to complete a survey identical to that used in our previous SZ classifier study. The answers were collected face to face. When necessary, trained assistants helped them complete the survey. These assistants provided only technical help and did not influence the answers. Notably, all participant diagnoses in this study were provided by experienced psychiatrists who had access to comprehensive treatment records, ensuring the sample quality for external validation. In total, we collected 150 samples from 61 schizophrenia patients, 56 MDD patients, 32 BD patients, and 1 OCD patient. Using these samples, we assessed the external validity of the SZ classifier. The primary reason for selecting MDD, BD, and OCD as the control group is that these disorders are theoretically easier to distinguish from schizophrenia, despite often presenting with overlapping clinical features that require careful differential diagnosis. The study design is also considered to possibly simulate the complexities encountered in actual clinical scenarios.

The study was conducted in accordance with the Declaration of Helsinki, and the protocol was approved by the Bioethics Review Committee of Fujita Health University (HM21-408).

### 2.3. Statistical Analysis

Statistical analyses in the current study were performed using Python (version 3.7; Python Software Foundation). The computational code editor used was the Jupyter Notebook 7 (Project Jupyter).

## 3. Results

Of the 61 schizophrenia patients, 46 were accurately classified, and the sensitivity of the model reached 75%. Among the 89 non-schizophrenia patients, 50 were incorrectly classified as having schizophrenia (Table 1). Of the 56 MDD patients, 31 (55%) were incorrectly classified as having schizophrenia, and of the 32 BD patients, 19 (59%) were incorrectly classified as having schizophrenia (Table 2).

## 4. Discussion

This study conducted an external validation of our predeveloped SZ classifier using outpatient data. The SZ classifier achieved a sensitivity of 0.75 (46 out of 61 patients) when applied to individuals diagnosed with schizophrenia. However, when applied to patients diagnosed with BD or MDD, the misclassification rates were 59% (19 out of 32) and 55% (31 out of 56), respectively.

The sensitivity achieved in the external validation exceeded that of the internal test (0.56) [7]. This disparity may stem from the fact that the external validation sample set relies on diagnoses by clinicians and regular medical visits, which are deemed more reliable than self-reported diagnoses obtained through online surveys. Hohmann emphasized that the dependency of the output is reliant on the accuracy of the big data and the sample size [14]. The model performance is limited by poor data quality and insufficient confounding variables [14]. Moreover, the sample set used for developing the SZ classifier was imbalanced. We adopted a well-defined algorithm—Synthetic Minority Over-sampling Technique (SMOTE)—to solve this problem [15]. Nevertheless, SMOTE does not always work perfectly when solving such a problem. Hence, it is reasonable to speculate that if the data used for developing the model were of higher quality, the performance of the SZ classifier would likely improve.

The SZ classifier demonstrated a misclassification rate of >50% when applied to MDD and BD patients. This could be attributed to the similarities between schizophrenia, MDD, and BD. The top five features, ranked according to their importance, of the SZ classifier were the frequency of sleep medication use, age, household income, type of employment, and bedtime [7]. Table S1 lists the characteristics of the external validation sample set based on these five features. The distributions between schizophrenia and MDD, as well as between schizophrenia and BD, were similar (see Supplementary Materials, Table S1). Clinically, MDD, BD, and OCD often present with symptoms similar to those of schizophrenia; thus, a careful differential diagnosis is required. Since our SZ classifier was not built using samples of MDD, BD, and OCD, the poor specificity observed in the external validation results was anticipated. 

Although the SZ classifier achieved a respectable sensitivity of 0.75, the misclassification rate exceeding 50% indicates that the model still requires significant improvements before it can be widely promoted across various communities or populations. We found no prior studies discussing external validation for machine learning models in schizophrenia classification. However, some relevant research based on machine learning techniques could be referenced and utilized. For instance, Bae used content from social media and compared four machine learning methods using natural language processing techniques for classifying schizophrenia [16]. The Random Forest model obtained the best AUC of 0.97, while Bae’s study did not involve artificial neural networks. Bracher-Smith trained schizophrenia classification models using genetic and demographic factors from the UK Biobank, where the neural network model achieved an AUC of only 0.67 [17], which is lower than the 0.86 AUC from our study (internal validation). Shanarova designed a machine learning-based diagnostic for schizophrenia using event-related potential data, achieving a sensitivity and specificity of 91% and 90.8%, respectively [18]. Overall, the prospects for machine learning in schizophrenia research are promising, primarily because it can analyze speech, behavior, image, and people’s creativity [19]. It has been shown that various machine learning-based models can assist specialists in predicting and diagnosing schizophrenia using medical history, genetic data, and even epigenetic information [19].

The primary advantage of using machine learning to construct a classification model is its ability to extract valuable information from extensive datasets, particularly those related to feature engineering, without explicit instructions [20]. For example, machine learning techniques have great potential for quantitatively distinguishing the differences among similar feature expressions. We have previously reported that schizophrenia patients have an increased likelihood of developing diabetes, cardiovascular disease, worsening of metabolic syndrome, depression, and sleep disorders [8]. Similar findings were reported for MDD [21]. Additionally, schizophrenia patients are observed to exhibit elevated rates of smoking, drinking, and drug consumption [8], and a similar pattern is also observed in MDD and BD patients [21,22]. Schizophrenia patients typically exhibit lower health literacy [8], while older BD patients experience a faster decline in health perception [23]. However, previous studies have rarely addressed the distinctions among these similar feature expressions for those diseases. Given that our survey questionnaire delved into the aforementioned issues (namely, the information was collected quantitatively), we have reason to believe that introducing samples of MDD, BD, and OCD during the model development could improve the SZ classifier’s performance. 

This study had several limitations. First, the sample set used for the external validation was collected only from Central Japan and may not be representative of the broader population. Additionally, the sample size was limited. Future studies should include more patients from diverse regions, as well as healthy controls, to enhance the reliability. Second, biases may arise from the online study design. Some individuals may not use the internet frequently, and patients with severe illnesses may struggle to complete the questionnaire owing to difficulties in concentration. However, in light of the changes in people’s habits following the Coronavirus pandemic in recent years, online methods such as apps have become increasingly applicable for conducting epidemiological research [24]. Third, relying on nonpsychiatric symptoms to detect the presence of mental disorders may seem arbitrary, despite yielding favorable results. Therefore, the findings of such studies should be interpreted and promoted with caution.

## 5. Conclusions

The SZ classifier currently faces challenges in accurately determining the presence or absence of schizophrenia at the individual level. The performance of the SZ classifier would likely improve if the data used during model development were of higher quality. Prior to widespread practical implementation, enhancements are necessary to bolster the accuracy and diminish misclassification rates. Considering the results of external validation and the inherent characteristics of machine learning techniques, there exists significant potential to improve the performance of the SZ classifier if samples of multiple diseases are included during the model development phase.

## Figures and Tables

**Table 1:** Confusion matrix of external validation for the SZ classifier: results of all cases.

		Observed Cases	
		Schizophrenia	Major Depressive Disorder, Bipolar, and Obsessive-Compulsive Disorder
Predicted cases	Schizophrenia	46	50
	Major depressive disorder, bipolar, and obsessive-compulsive disorder	15	39
Total		61	89
Sensitivity		0.75	–
Specificity		–	0.44

**Table 2:** Confusion matrix of external validation for the SZ classifier: results of the non-schizophrenia cases.

		Observed Cases		
		Major Depressive Disorder	Bipolar	Obsessive-Compulsive Disorder
Predicted cases	Schizophrenia	31	19	0
	Non-schizophrenia	25	13	1
Total		56	32	1
Misclassification rate		0.55	0.59	0

## Data Availability

The data presented in this study are available from the corresponding author upon reasonable request and after appropriate procedures, including approval from the institutional ethics review committee.

## Supplementary Materials

The following supporting information can be downloaded at https://www.mdpi.com/article/10.3390/jcm13102970/s1. References [25,26,27,28,29,30,31,32,33,34,35,36] are cited in the supplementary materials.
